# Imprints of independent allopolyploid formations on patterns of gene expression in two sibling yarrow species (*Achillea*, Asteraceae)

**DOI:** 10.1186/s12864-021-07566-6

**Published:** 2021-04-13

**Authors:** Duo Chen, Peng-Cheng Yan, Yan-Ping Guo

**Affiliations:** 1 Key Laboratory of Biodiversity Science and Ecological Engineering of the Ministry of Education, and College of Life Sciences, Beijing Normal University, Beijing, China; 2 Beijing Tangtang Tianxia Biotechnology Co., Ltd, Beijing, China

**Keywords:** Allopolyploid speciation, RNA-sequencing, Inheritance of gene expression, Homeolog express bias, *Achillea*

## Abstract

**Background:**

Polyploid species often originate recurrently. While this is well known, there is little information on the extent to which distinct allotetraploid species formed from the same parent species differ in gene expression. The tetraploid yarrow species *Achillea alpina* and *A. wilsoniana* arose independently from allopolyploidization between diploid *A. acuminata* and *A. asiati*ca. The genetics and geography of these origins are clear from previous studies, providing a solid basis for comparing gene expression patterns of sibling allopolyploid species that arose independently.

**Results:**

We conducted comparative RNA-sequencing analyses on the two *Achillea* tetraploid species and their diploid progenitors to evaluate: 1) species-specific gene expression and coexpression across the four species; 2) patterns of inheritance of parental gene expression; 3) parental contributions to gene expression in the allotetraploid species, and homeolog expression bias. Diploid *A. asiatica* showed a higher contribution than diploid *A. acuminata* to the transcriptomes of both tetraploids and also greater homeolog bias in these transcriptomes, possibly reflecting a maternal effect. Comparing expressed genes in the two allotetraploids, we found expression of ca. 30% genes were species-specific in each, which were most enriched for GO terms pertaining to “defense response”. Despite species-specific and differentially expressed genes between the two allotetraploids, they display similar transcriptome changes in comparison to their diploid progenitors.

**Conclusion:**

Two independently originated *Achillea* allotetraploid species exhibited difference in gene expression, some of which must be related to differential adaptation during their post-speciation evolution. On the other hand, they showed similar expression profiles when compared to their progenitors. This similarity might be expected when pairs of merged diploid genomes in tetraploids are similar, as is the case in these two particular allotetraploids.

**Supplementary Information:**

The online version contains supplementary material available at 10.1186/s12864-021-07566-6.

## Background

Polyploidy is an important mechanism of plant speciation. In an allopolyploid species, the combined effects of two or more diverged subgenomes and their regulatory interactions can lead to a myriad of genetic and epigenetic modifications described as genomic and transcriptomic shock [1–6]. The resulting changes in gene expression may often generate phenotypic variation affecting individual fitness and evolution of allopolyploids [7–13]. Analyses of synthetic polyploid plants have demonstrated that genomic and transcriptomic shock usually occurs immediately after polyploidization [1, 14–17], though changes may also take place during later stages of the evolutionary history of a polyploid species [3, 6, 18, 19].

Polyploid species often consist of lineages that originated independently and recurrently from the same parental species [20, 21]. Such recurrent formation can result in karyotypic, genomic, transcriptomic and phenotypic variation across lineages as demonstrated in recently originated allotetraploid species of *Tragopogon* (Asteraceae) [22–27]. However, whereas different lineages of the same allopolyploid species have been studied in detail, divergent species derived by independent origins from the same parental species have been reported less frequently and studied less [28–30]. Only in the orchid genus, *Dactylorhiza*, has research been conducted on gene expression and epigenetic differences among sibling allotetraploids derived from the same parental species pair. This showed that both kinds of differences occurred and were stable among these allotetraploid species, raising the possibility that they reflect divergent adaptation to the different environmental conditions experienced by the species [28, 29].

To shed further light on how gene expression might differ between allopolyploid species that originated independently from the same progenitor species, we focus here on two allotetraploid yarrow species, *Achillea alpina* L. and *A. wilsoniana* Heimerl ex Hand. -Mazz., and their parental species, *A. acuminata* (Ledeb.) Sch. -Bip. and *A. asiatica* Serg. (Asteraceae). In China, these tetraploid species have different distributions, with *A. alpina* occurring in the northeast and *A. wilsoniana* in the southwest of the country [30, 31]*.* Our previous research indicated that the two tetraploids originated independently 35–80 kya following hybridization between their diploid parents during the megainterstadial before the Last Glacial Maximum. Two independent contacts between the parental species were involved, possibly in deglaciated habitats located near refugia present in the mountains of northeast China and relatively southwestern in the Qinling Mountains, respectively [30]. According to plastid sequencing data, *A. asiatica* mostly likely acted as the maternal parent of both tetraploids [32, 33].

To investigate transcriptome changes occurred during allopolyploidization and the following long-term evolution, it is not only necessary to check specific and coexpressed genes among progeny and progenitor species, but also to examine total and relative expression levels of homeologous genes in allotetraploids. Relative expression levels of homeologs may reflect preexisting parental relative levels (parental legacy) or originate following allopolyploidy with one homeolog preferentially expressed relative to the other (expression bias) [34–37]. *Achillea alpina* and *A. wilsoniana* are ideal for such analysis for the following reasons. First, their parental-offspring relationships are clear and simple (no complicated reticulate relationships are involved according to previous studies). Second, the parental species are extant, making it feasible to compare data from allopolyploids with that of their progenitors. Third, the progenitor species, *A. acuminata* and *A. asiatica*, show high levels of genomic sequence divergence [32, 38], while each allopolyploid species maintains both parental genomes intact, having experienced only low levels of homeologous recombination [30, 32, 33]. For these reasons, it is easy to distinguish homeologous genes from each other in the allopolyploid transcriptome, and to measure parental contributions and homeolog expression bias.

In this study, we screened the transcriptome profiles of the two *Achillea* allotetraploid species and their diploid progenitor species by means of whole transcriptome sequencing. By a comparative analysis of these transcriptomes, we examined first the inheritance of parental gene expression, and second relative parental contributions and homeolog expression bias. From our results, we ask whether parental effects which are frequently found in plant hybrid/allopolyploid transcriptomes, are apparent in the present polyploid system. Furthermore and most importantly, we question to what extent inherited patterns of gene expression are similar in different allopolyploids derived from the same parental species, and how significant evolutionary factors, e.g. natural selection and/or genetic drift, have influenced divergent gene expression profiles of the two independently evolved tetraploid species.

## Results

### Transcriptome profiles

Approximately 34–49 million 100 bp paired-end raw reads were generated for a library of each of the studied *Achillea* species. After removing adapter sequences and filtering out reads with low quality, 93.2–96.4% of clean reads were obtained (Table S1). The initial transcripts were assembled and filtered to 51,414–88,150 unigenes across the studied species, with the N50 length of unigenes always longer than the average length of unigenes in each sample (Table 1). The proportion of unigenes with complete or partial ORFs was 63–71%. These unigenes were used for subsequent gene expression analysis (Table 1). The FPKM values of unigenes showed that data correlation among biological replicates of the same tissue/organ of a species/population was higher than among different tissues/organs, indicating that experimental sampling was repeatable and reliable (Fig. S1).

**Table 1:** Information of unigenes in the present RNA-Seq data

	acuARX	acuQL	asi	alp	wil
Number of assembled transcripts by Trinity	177,816	180,194	272,030	282,619	300,158
Number of unigenes	51,414	55,391	59,600	81,143	88,150
Average length of unigenes (bp)	1230.20	1243.32	1074.86	976.40	1011.16
N50 length of unigenes (bp)	1678	1687	1544	1386	1432
Number of lncRNAs	5794	5794	7604	11,409	13,748
Number of unigenes with no ORF	9229	11,094	10,342	15,078	18,603
Number of unigenes with complete ORF	21,801	24,123	20,997	24,412	27,984
Number of unigenes with partial ORF	14,590	14,380	20,657	30,244	27,815

Abbreviation of accession names: acuARX for Arxan population of *A. acuminata*; acuQL for Qinling population of *A. acuminata*; alp for *A. alpina*; asi for *A. asiatica*; wil for *A. wilsoniana*

### Specifically expressed and coexpressed genes among each allotetraploid species and its diploid progenitors

**Fig. 1:**
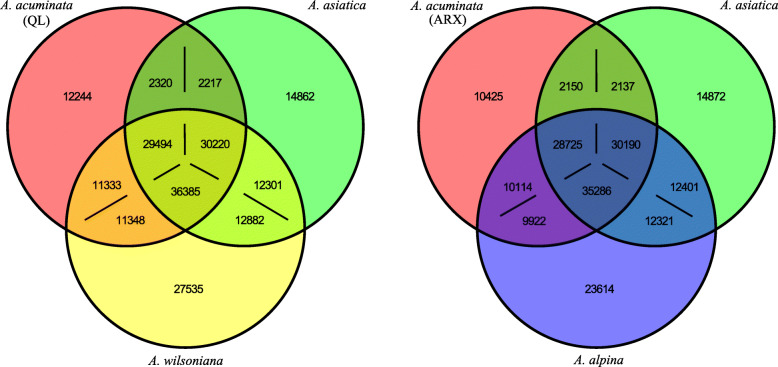
Venn diagrams showing amounts of coexpressed and specifically expressed genes of the studied allotetraploid species and their diploid progenitors. As the two allopolyploid species originated independently in different regions, and as the diploid *A. acuminata* shows population genetic differentiation, the analysis was conducted separately for each tetraploid species. In the coexpressed gene category, gene number in each species is given (copy-number on some loci may be different among species). Abbreviations: ARX, Arxan Mt.; QL, Qinling Mts

As shown in the Venn diagrams (Fig. 1), there were 23,614 (29.1%) and 27,535 (31.2%) genes showing species-specific expression in the allotetraploids *A. alpina* and *A. wilsoniana*, respectively, equating to higher proportions than in the diploid parental species (20–25%) and indicating rather high amounts of novel gene expression in both allotetraploids. The numbers of genes expressed in both parents, but not detected in the allotetraploid transcriptome, were 2150/2137 and 2320/2217 in *A. alpina* and *A. wilsoniana*, respectively, suggesting a relatively low level of gene silencing or loss. With regard to coexpression of genes, 35,286 unigenes (about 43.5% of all unigenes) were coexpressed between *A. alpina* and both diploid species, and 36,385 (about 41.3% of all the unigenes) were coexpressed by *A. wilsoninana* and the two diploids (Fig. 1).

**Fig. 2:**
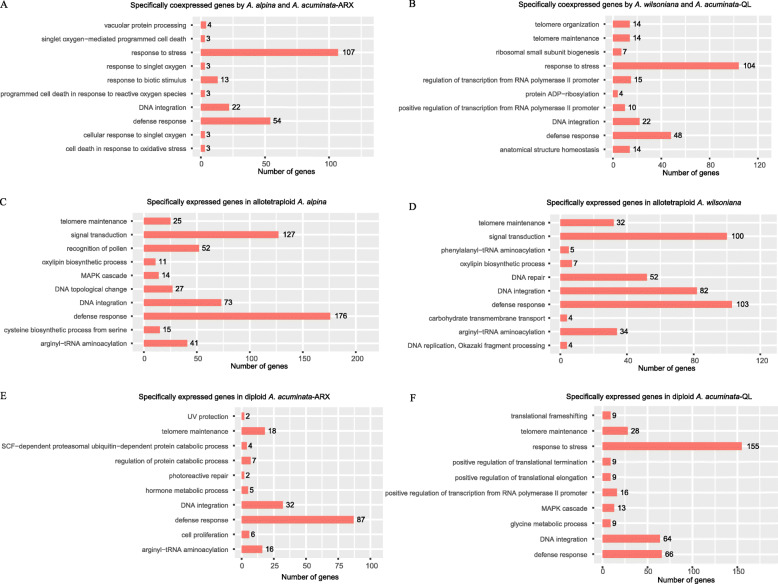
The top ten most enriched GO terms related to biological process (BP) of specifically coexpressed genes of each allotetraploid species with its sympatric population of *A. acuminata* (**a & b**), species-specific expressed genes in a comparison between the two allotetraploid species (**c & d**), and population-specific expressed genes in a comparison between two populations of diploid *A. acuminata* (**e & f**) (*P*-value < 0.05). These data suggested that the specifically expressed genes were mostly enriched in gene classes pertaining to biological response to environment. The full information of enriched GO terms are listed in Additional Files 6 and 7. Abbreviations: ARX, Arxan population of *A. acuminata*; QL, Qinling population of *A. acuminata*

Particularly interesting are the genes of each tetraploid specifically coexpressed with each parental species as this indicates the relative contribution of each parent to the transcriptome of each tetraploid. We found that *A. alpina* specifically coexpressed 9922 unigenes with diploids *A. acuminata*, and 12,321 unigenes with *A. asiatica*; while *A. wilsoniana* coexpressed 11,348 and 12,882 unigenes with *A. acuminata* and *A. asiatica*, respectively (Fig. 1). Thus, both tetraploids coexpressed more genes with *A. asiatica* than with *A. acuminata*. Gene Ontology (GO) analysis indicated significant enrichment of these coexpressed genes mostly in terms “response to stress” and “defense response”, suggesting that the tetraploid species inherited environmental response genes separately from both progenitors (Fig. 2: A, B; Additional file 6).

### Species-specific and coexpressed genes in the two allotetraploid species

Table 2 shows that comparing the expressed genes in the tetraploids, 29.4% genes expressed in *A. alpina* showed species-specific expression and 33.9% genes expressed in *A. wilsoniana* were species-specific. Among the coexpressed genes, 78%–83% were expressed equally in both species, while only about 10% showed up- or down-regulation in one or the other (Table 2). Most enriched GO terms related to biological process (BP) of genes exhibiting species-specific expression pertained to “defense response” in both tetraploids (Fig. 2: C, D; Additional file 7).

**Table 2:** Number of specifically and differentially expressed genes in the two studied allotetraploid species

Specific in ***A. alpina***	Specific in ***A. wilsoniana***	Expressed in both tetraploids (stem apex)	Expressed in both tetraploids (leaf)
23845 (29.4%)	29881 (33.9%)	4049 (up-regulate in *A*. *alpina*)	2879 (up-regulate in *A*. *alpina*)
		8328 (up-regulate in *A*. *wilsoniana*)	6727 (up-regulate in *A*. *wilsoniana*)
		44524 (equal expression in both)	47267 (equal expression in both)

In parallel, we found approximately 30% of genes showing population-specific expression in diploid *A. acuminata*; these were most enriched for GO terms pertaining to “defense response” and/or “response to stress” (Fig. 2: E, F; Additional file 7). Moreover, genes coexpressed by each tetraploid with its sympatric *A. acuminata* population were also most enriched for GO terms related to “response to stress” and “defense response” (Fig. 2: A, B; Additional file 6]). These results imply that the two geographically separated tetraploids may have inherited genes and expression patterns from their sympatric diploid parental species which could be important in local adaptation.

### Inheritance patterns of gene expression

**Fig. 3:**
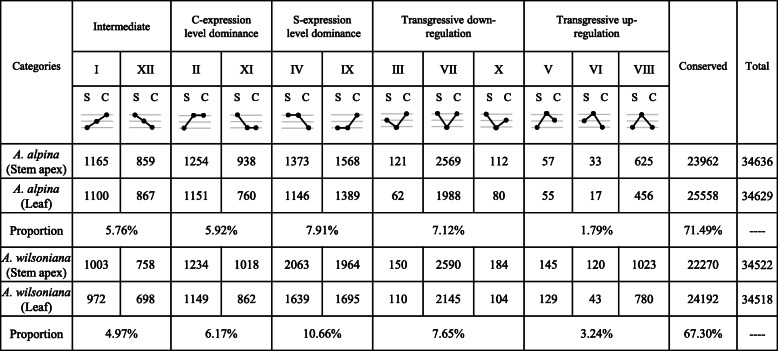
Inheritance categories of gene expression of the studied allotetraploid species. The categorization involving 12 states of differential expression (labeled with Roman numeral I–XII) is modified from Rapp et al. (2009) [39]. A cartoon depiction is provided for each of the 12 states, where parental states (S for *A. asiatica*; C for *A. acuminata*) are on the outer edges and the allotetraploid is in the middle. Dots on the same horizontal line indicate statistically equal expression level, whereas dots on higher or lower horizontal lines refer to significantly higher or lower expression level. The ‘Intermediate’ states, I and XII, indicate gene expression levels in the allopolyploid being significantly different from, but intermediate between the parental levels. The ‘conserved’ refers to genes with basically equal expression levels among the allotetraploid and both parental species. The number of genes of each category is given, and the percentage of each category group in all expressed genes is provided

Figure 3 shows the numbers and proportions of differentially expressed genes (DEGs) among all expressed genes in the allotetraploids. Most of these genes (71.49% in *A. alpina* and 67.30% in *A. wilsoniana*) were ‘conserved’, meaning that the total expression of homeologs for a given gene in the allotetraploids was statistically similar to the expression levels of that gene in both parental species.

Altered gene expression in the tetraploids was evidenced by expression inheritance patterns classified into 12 categories. Thus, 5.8 and 5.0% of expressed genes in *A. alpina* and *A. wilsoniana*, respectively, had expression levels intermediate to the parental species (categories I and XII in Fig. 3). Approximately 15% of genes showed “expression-level dominance” (categories II, XI, IV and IX) with both tetraploids exhibiting greater *A. asiatica* expression-level dominance (S-dominance) than *A. acuminata* dominance (C-dominance) (categories IV and IX vs. II and XI). Finally, both tetraploids possessed more transgressively downregulated genes (categories III, VII and X) than transgressively upregulated genes (categories V, VI and VIII).

### Relative homeolog contribution and homeolog expression bias

The two allotetraploids displayed a relatively small proportion of silent/lost parental genes. Moreover, they exhibited imbalanced silencing/loss of homeologs between the two parental subgenomes. Silence/loss of genes were more evident for *A. acuminata*-homeologs than for *A. asiatica*-homeologs, implying preferential expression of the *A. asiatica*-subgenome in both tetraploids (Table 3).

**Table 3:** Number of silent/lost homeologs in the studied allotetraploids

Samples	Number of silent/lost *A. asiatica-*homeologs (%)	Number of silent/lost *A. acuminata-*homeologs (%)
*A. alpina* (Stem apex)	362 (3.60%)	539 (5.36%)
*A. alpina* (Leaf)	311 (3.67%)	479 (5.65%)
*A. wilsoniana* (Stem apex)	370 (3.50%)	517 (4.89%)
*A. wilsoniana* (Leaf)	331 (3.99%)	417 (5.02%)

**Fig. 4:**
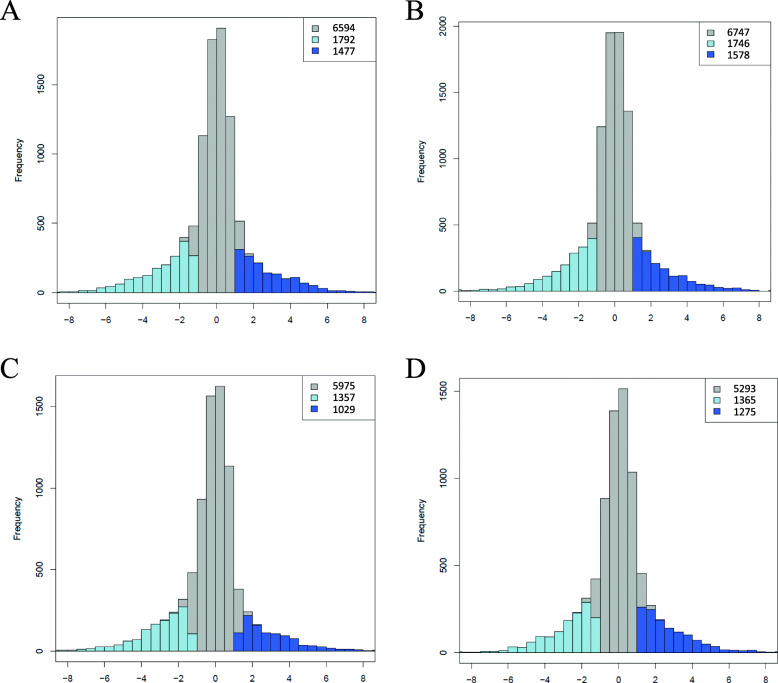
Histograms showing relative expression levels of homeologous genes in the studied allotetraploid transcriptomes. **a, b** Data from stem apex. **c, d** Data from leaf tissue. **a, c** for *A. alpina*; **b, d** for *A. wilsoniana*. The abscissa is Rh [log_2_ (acu-homeolog/asi-homeolog)], and the ordinate is the number of homeolog pairs. Gray columns correspond to homeolog pairs with equal expression level of two parental copies; light blue columns correspond to homeolog pairs with higher expression of the *A. asiatica*-copy, and vise versa, dart blue columns correspond to homeolog pairs with higher expression of the *A. acuminata*-copy (P-value < 0.05, FDR < 0.05). Numbers at the upper right corner indicate the number of homeolog pairs of each category

The relative homeolog contribution to total expression levels of allotetraploid genes was quantified by Rh [Rh = log_2_ (acu-homeolog/asi-homeolog)] (Fig. 4). Approximately two-thirds of homeolog pairs displayed equal expression of parental copies, and the remaining one-third exhibited different expression levels of parental homeologs. Among the differentially expressed homeologous pairs, more exhibited higher expression of the *A. asiatica* copy than the *A. acuminata* copy.

**Fig. 5:**
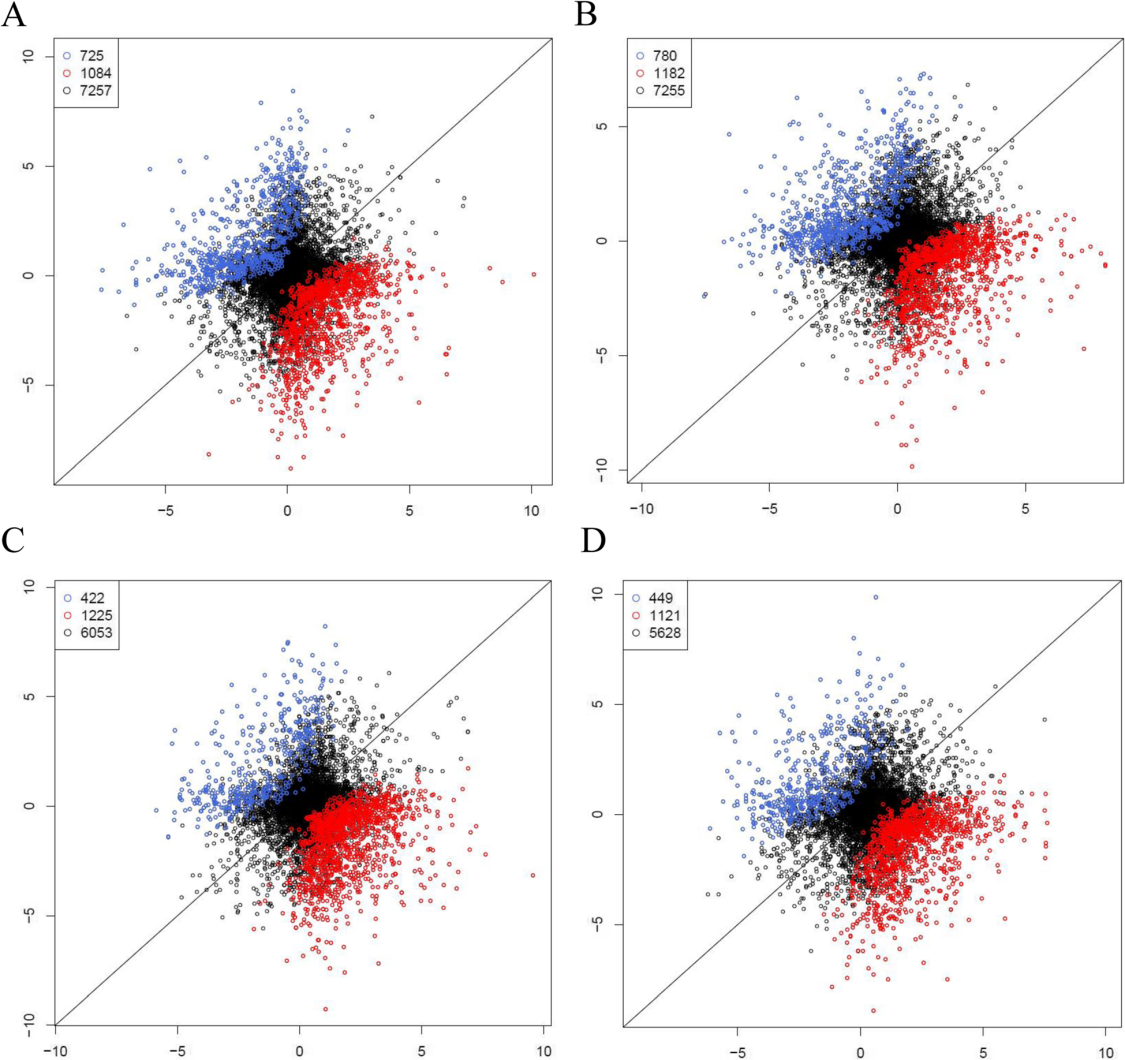
Scatter plots showing expression bias of homeologs in the studied allotetraploid transcriptomes. **a, b** Data from stem apex. **c, d** Data from leaf tissue. **a, c** for *A. alpina*. **b, d** for *A. wilsoniana*. The abscissa is Rp [log_2_ (*A. acuminata*/*A. asiatica*)], and the ordinate is Rh [log_2_ (acu-homeolog/asi-homeolog)]. Black circles correspond to homeolog pairs without expression bias; blue circles correspond to the homeolog pairs with expression bias toward *A. acuminata* (namely C-bias); and red circles correspond to homeolog pairs with biased expression of the *A. asiatica*-copy (namely S-bias) (P-value < 0.05, FDR < 0.01, Fisher’s exact text). Numbers at the upper left corner indicate the number of homeolog pairs of each category

To determine if the detected differential expression of homeologs is derived from pre-existing differences in parental gene expression levels, or is due to homeolog expression bias, we compared Rh with the relative expression of orthologs between the parental species, Rp [Rp = log_2_ (*A. acuminata*/*A. asiatica*)] (Fig. 5). Approximately 79% of homeolog pairs in the tetraploids displayed vertical inheritance of pre-existing parental expression levels, that is, without expression bias. Among the remaining 21% homeolog pairs that displayed parental expression bias, S-bias (bias toward *A. asiatica* copy) was more common than C-bias (bias toward *A. acuminata* copy).

To understand the possible influence of expression bias to the relative contribution of the parental homeologs, we integrated data sets of relative homeolog expression level and homeolog bias (Table S2). Of the homeolog pairs showing equal expression of parental copies, 35% showed expression bias, while the rest simply maintained pre-existing parental expression levels. Of the homeolog pairs with unequal expression levels, most might have resulted from homeolog expression bias. For instance, out of 1396 homeolog pairs showing higher expression level of the *A. asiatica* copy, 1037 (74.3%) displayed expression bias toward the *A. asiatica* copy (Table S2).

### Validation of RNA-Seq analysis by RT-qPCR

To validate the analysis and data obtained by RNA-sequencing, differential expression of genes was checked using RT-qPCR assays. Unigenes exhibting different inheritance patterns of gene expression (intermediate expression, *A. acuminata*/*A. asiatica* expression-level dominance, transgressive expression) were randomly chosen for RT-qPCR verifying. For all 10 unigenes tested, expression patterns revealed by qRT-PCR assays were consistent with those evident in the RNA-Seq data (Fig. S2), demonstrating the reliability of data produced by RNA-sequencing.

## Discussion

To understand the influence of hybridization and polyploidy on the inheritance of gene expression from parental to allopolyploid species, we conducted a transcriptome analysis on two allotetraploid *Achillea* species that originated independently from the same two parental species. We evaluated RNA-Sequencing data to determine: (*i*) species-specific gene expression and coexpression among both tetraploid and progenitor diploid species; (*ii*) inheritance patterns of parental gene expression; and (*iii*) parental contribution to gene expression level in the tetraploids, and occurrence of homeolog expression bias.

### Gene expression profiles in the allotetraploid species with influence of maternal effect

Both hybridization and polyploidization can alter gene expression between progenitors and allopolyploid offspring by affecting the number of expressed genes and their expression levels. In the present analysis only 3.6%–4.7% (Fig. 1) genes expressed in the diploids were not detected in the tetraploid species, suggesting a low level of gene silencing (or loss). On the other hand, each of the tetraploid species possessed a high proportion (approximately 30%) of species-specific expressed genes (23,614 out of 81,143 genes in *A. alpina* and 27,535 out of 88,150 genes in *A. wilsoniana*, Fig. 1), suggesting that hybridization and polyploidy activate some genes not expressed in the diploids.

In hybrid plants, maternal effects may have a strong influence on morphological, life-history and physiological traits, which can be beneficial if the maternal phenotype is linked to increased fitness [40–43]. The present study showed that global gene expression of both *Achillea* tetraploids was frequently more similar to *A. asiatica* than to *A. acuminata*, as reflected by the number of coexpressed genes between species, expression-level dominance, relative homeolog contribution, homeolog-specific expression and homeolog expression bias. This similarity to *A. asiatica* suggests a maternal effect on gene expression with both tetraploids previously shown to have had an *A. asiatica*-like ancestor as their maternal parent [32, 33].

It has been suggested that parental expression-level dominance in allopolyploids mainly results from up- or down-regulation of one of the homeologous copies, usually of the ‘less dominant’ parent [44, 45]. Homeolog expression bias may lead to higher expression of one of the parental gene copies due possibly to a difference between parental subgenomes in number and distribution of transposable elements (usually repressing nearby genes), mismatches between parental copies of *trans*-elements and their target genes, and persistent epigenetic resetting [6, 36, 37, 46, 47]. Maternal effects resulting from one or more of these causes have been reported previously in a number of allopolyploids, e.g. *Gossypium hirsutum* [18, 48], *Spartina anglica* [49], *Triticum aestivum* [50] and *Tragopogon miscellus* [51].

### Comparative global gene expression patterns of allopolyploids independently derived from the same parent species

Previous research on *Dactylorhiza* showed that three sibling allotetraploid species derived from the same two parental species were divergent epigenetically and in gene expression, and it was suggested that these differences may have been important in their adaptation to different habitats [28, 29]. Similarly, the two *Achillea* tetraploids studied here originated independently due to multiple contacts between the same two parental species in different geographical regions, with population genetic analysis showing them to be genetically well-differentiated [30]. Comparing expressed genes in the two tetraploids, we found a high proportion of species-specific expression (29.4% in *A. alpina* and 33.9% in *A. wilsoniana*) (Table 2). These species-specific expressed genes were enriched for GO terms pertaining to “defense response” (Fig. 2; Additional file 7). Polyploidy may confer adaptive novelties, as indicated in the aforementioned orchids and in *Achillea* [9, 13, 28, 29, 39, 52–54]. Species-specific and differential expression of genes of *A. alpina* and *A. wilsoniana* might have partly originated as a result of the independent allopolyploidization events that gave rise to these two species, but also to independent post-speciation events due to natural selection and/or genetic drift.

Despite the species-specific and differentially expressed genes between the two allotetraploids, they display similar transcriptome changes in comparison to their diploid progenitors, e.g., maternal effects of *A. asiatica* have influenced both tetraploid transcriptomes as suggested by inheritance patterns of gene expression, parental contributions to tetraploid transcriptomes, and homeolog expression bias.

## Conclusion

The present comparative transcriptome analysis revealed that two independently originated *Achillea* allotetraploid species exhibited difference in gene expression, some of which was inevitably produced by randomly combined effects of hybridization and polyploidization, but some others must have occurred and maintained under natural selection and/or genetic drift during their tens of thousand years of evolution [30]. Particularly, the species-specific expressed genes enriched for GO terms pertaining to “defense response” suggested differential adaptation during their post-speciation evolution. On the other hand, they showed similar transcriptome changes in comparison to their diploid progenitors. This similarity may be expected when the combinations of genomes merged by different allopolyploidization events were similar [37]. More detailed studies are now required to determine the adaptive significance of differences in gene expression between these two allotetraploid species, which have been revealed by our analysis.

## Methods

### Plant materials

Plants used for this study were grown in laboratory incubators (16 h: 8 h light-dark cycles at 23 °C) for 3–4 months from achenes collected from natural populations of the four *Achillea* species in China. Achenes of the allotetraploid *A. alpina,* were sampled from Arxan Mountain in the northeast (N 47°17′, E 120°27′; 860 m), where both diploid species also occur in sympatry. Achenes of the other tetraploid, *A. wilsoniana* in the southwest, were collected from Taibai Mountain (N 33°59′, E 107°17′; alt. 2094 m) in Qinling mountain range, approximately where this allotetraploid was originated. Because populations of diploid species *A. acuminata* in NE China and in Qinling mountains are genetically differentiated [30], achenes of this species were collected from both Arxan Mt. and Taibai Mt.. In contrast, we collected achenes of diploid *A. asiatica* only from Arxan Mt. as populations of this species across E Asia are genetically similar [30].

Tissues analyzed were stem apex and the first fully-spread leaf beneath the stem apex. Samples of different tissues were separately snap frozen in liquid nitrogen and stored at − 80 °C. Three replicates of each tissue were obtained, with each replicate containing samples pooled from several plant individuals so that there was sufficient RNA for analysis.

### RNA extraction, cDNA library construction and RNA sequencing

Total RNA was extracted using a RNeasy Plus Mini Kit (Qiagen, Hilden, Germany). RNA concentration and quantification were determined using the NanoDrop 2000 spectrophotometer (Thermo Scientific, USA). RNA-sequencing libraries of each sample were constructed and sequenced on an Illumina Hiseq 2000 platform with 100 bp paired-end reads by the Biodynamic Optical Imaging Center (BIOPIC) of Peking University (Beijing, China). The sequencing data have been deposited with links to BioProject accession number PRJNA669168 in the NCBI BioProject database (https://www.ncbi.nlm.nih.gov/bioproject/PRJNA669168).

### RNA-Seq de novo assembly and annotation

The number and quality of raw reads from each library were evaluated with FastQC v. 0.11.2 (http://www.bioinformatics.babraham.ac.uk/projects/fastqc). Adapter sequences, low quality bases (Q < 20) and unknown nucleotides (Ns) were trimmed using Trimmomatic v. 0.32 [55]. After trimming, both end of reads with length above 25 bases were kept for assembly. To minimize technical bias, all filtered clean reads from three biological replicates were used to conduct de novo transcriptome assembly by Trinity (v. r2013-02-25) [56]. Redundant transcripts were removed using CD-HIT (v. 4.6) [57] with 90% identity, and the longest transcript in each group was retained. Positively expressed genes were defined using an empirical cutoff value (FPKM > 1), and those with more than 200 bp were chosen as reliable unigenes [58]. The recognition of ORFs (open reading frames) and lncRNA were conducted on unigenes by TransDecoder (v. 2.0.1) (http://transdecoder.github.io/) and CNCI, separately, and unigenes with a complete or partial ORF were prepared for subsequent gene expression analysis [59].

Functions of unigenes were identified by searching against NCBI NR databases using locally installed BLASTX with an E-value cutoff of 1e-5, and the best alignment results were assigned as annotations of unigenes. The same strategy was applied to searches in Uniprot and KEGG databases.

### Ortholog and homeolog identification

All unigene sequences were aligned using BLASTN with cutoff E-value of 1e-10, with orthologous gene families identified by OrthoFinder (v. 0.4.0) [60, 61]. BLAST similarity searches were performed for pairwise comparisons between libraries. Orthologous genes were standardized by setting E-value ≤5e-100, alignment length ≥ 200 bp, and identity ≥90%.

Previous data showed that the two progenitors of the studied allotetraploids are genetically distinct, and the allotetraploid species have maintained their parental subgenomes relatively intact [30, 32, 33]. This made it easy to identify homeologous gene copies in the allotetraploid species using the single nucleotide polymorphisms (SNPs) between the two diploid species. Clean reads of diploid and allotetraploid species were mapped to the assembled unigenes of the two allotetraploids, and SNPs were identified by SAMtools (v0.1.17) [62]. Only SNPs that could tell the genomes of the parental species *A. acuminata* and *A. asiatica* apart were chosen, and clean reads in the allotetraploids exhibiting parental SNPs were parsed into homeolog-specific bins using custom perl scripts so that reads in the tetraploids were designated as of *A. acuminata-* or *A. asiatica*-type.

### Differential expression among species

Species-specific expressed and species-coexpressed genes were identified using orthologous gene families as units. Only genes coexpressed in the allopolyploid and both of parental species were further analyzed for gene expression levels. The number of clean reads mapped onto each gene was counted by RSEM (v. 1.1.13) [63] and the expression level of an unigene was determined as the average of three biological replicates. The analysis for differential expression between an allopolyploid and each of its diploid progenitors was performed using edgeR (v. 2.2.5) in R software (v. 2.13) with the trimmed mean of M-values (TMM) to normalize read counts within and across libraries. Benjamini and Hochberg (BH) methods were used to adjust *p*-values to account for significance of differentially expressed genes (DEGs) [64, 65]. DEGs were identified by absolute value of log_2_ (fold change) > 1 and FDR < 0.05 using a negative binomial test. DEGs among the allotetraploid and its diploid progenitors were assigned to 12 categories modified from Rapp et al. (2009) [48] containing intermediate expression of the polyploids between that of the parents, expression-level dominance, transgressive expression, and conserved (equal in all species).

### Analyses of homeolog expression bias

To calculate the expression levels of homeologs in the allotetraploid, read number mapped onto putative parental interspecific SNPs was counted and the average of those read number was calculated when more than one such SNP occurred in one fragment. To understand the homeolog-specific contributions to the allotetraploid gene expression, the analysis of differential expression was assessed between the two parental homeologs via a negative binomial test in edgeR package with the criterion of absolute value of log_2_ (fold change) > 1, FDR < 0.05 and *P*-value < 0.05. To further quantify expression level differences, we defined the relative expression of homeologs (Rh) as: Rh = log_2_ (acu-homeolog/asi-homeolog), where acu-homeolog or asi-homeolog is the expression level of the corresponding homeolog. This measurement can be computed for any homeolog pair with non-zero read counts (testable homeolog pairs); when Rh > 0, it indicates a higher expression level of the *A. acuminata*-homeolog than the *A. asiatica*-homeolog, and vice versa, when Rh < 0, the *A. asiatica*-homeolog may be expressed higher.

To examine the homeolog expression bias, we further defined the relative expression level of orthologous pairs of genes in parental species (Rp) as: Rp = log_2_ (*A. acuminata*/*A. asiatica*) and compared Rh with Rp using Fisher’s exact tests with the criterion of absolute value of log_2_ (fold change) > 1, FDR < 0.01 and P-value < 0.05. When Rh > Rp, it indicates expression bias toward diploid *A. acuminata*, and vice versa, when Rh < Rp, expression bias toward diploid *A. asiatica*.

### Validation of DEGs by reverse transcription real-time quantitative PCR (RT-qPCR)

To confirm the differential gene expression presented by the RNA-Seq data, we performed reverse transcription Real-Time quantitative PCR (RT-qPCR) analysis on several randomly selected genes. Gene-specific primer pairs were designed by the Primer Premier 5.0. Tissue/organ samples were the same as for the RNA-Seq analysis. Three independent batches of RNA were isolated as biological replicates. The Fast Quant RT kit (with gDNase) (Tiangen Biotech, Beijing, China) was used for cDNA synthesis following the manufacturer’s instructions. Then SYBR Premix Ex TaqTM (Tli RNaseH Plus) (Takara) was used for qPCR reactions. PCR reactions were performed on a 7500/7500 Fast Real-Time PCR System (Applied Biosystems) with the following program: 95 °C for 5 min, and then 40 PCR cycles at 95 °C for 5 s; 60 °C for 34 s. The glucose 6-phosphate dehydrogenase (G6PDH) and protein phosphatase 2A (PP2A) genes, which were confirmed having similar expression level among all the studied species by RNA-seq and qRT-PCR, were used as the internal reference genes. A relative quantitative method (delta-delta Ct) was used to evaluate the expression level of candidate genes. Primers used in this study are listed in Table S3.

## Supplementary Information

**Additional file 1: Supplementary Fig. S1.** Correlation analysis of transcriptome data from different samples.

**Additional file 2: Supplementary Fig. S2.** Patterns of gene expression detected by RT-qPCR to verify the RNA-Seq data.

**Additional file 3: Supplementary Table S1.** Information of reads in the transcriptome data.

**Additional file 4: Supplementary Table S2.** Relative expression level and expression bias of homeologs in the studied *Achillea* allotetraploid species.

**Additional file 5: Supplementary Table S3.** Primers used in RT-qPCR assays.

**Additional file 6. **Full information of enriched GO terms of genes specifically coexpressed by each allotetraploid species with each of its diploid progenitors as shown in Fig. 1. The top ten most enriched GO terms related to biological process (BP) of genes specifically coexpressed of each allotetraploid species with its sympatric diploid *A. acuminata* are shown in Fig. 2.

**Additional file 7. **Full information of enriched GO terms of (1) species-specifically expressed genes when the two allotetraploid species are compared, and (2) population-specifically expressed genes when two populaitons of the diploid parental *A. acuminata* are compared. The top ten most enriched GO terms related to biological process (BP) are shown in Fig. 2.

## Data Availability

The data generated and analyzed by this study have been deposited with links to BioProject accession number PRJNA669168 in the NCBI BioProject database (https://www.ncbi.nlm.nih.gov/bioproject/PRJNA669168).
