# Supplementary material for: Imprints of independent allopolyploid formations on patterns of gene expression in two sibling yarrow species (Achillea, Asteraceae)
Source: BMC Genomics. 2021 Apr 13;22:264. doi: 10.1186/s12864-021-07566-6 (PMC8045213; doi:10.1186/s12864-021-07566-6)
Supplement: Supplementary file 1 — Additional file 1: Supplementary Fig. S1. Correlation analysis of transcriptome data from different samples. [file 12864_2021_7566_MOESM1_ESM.pdf]

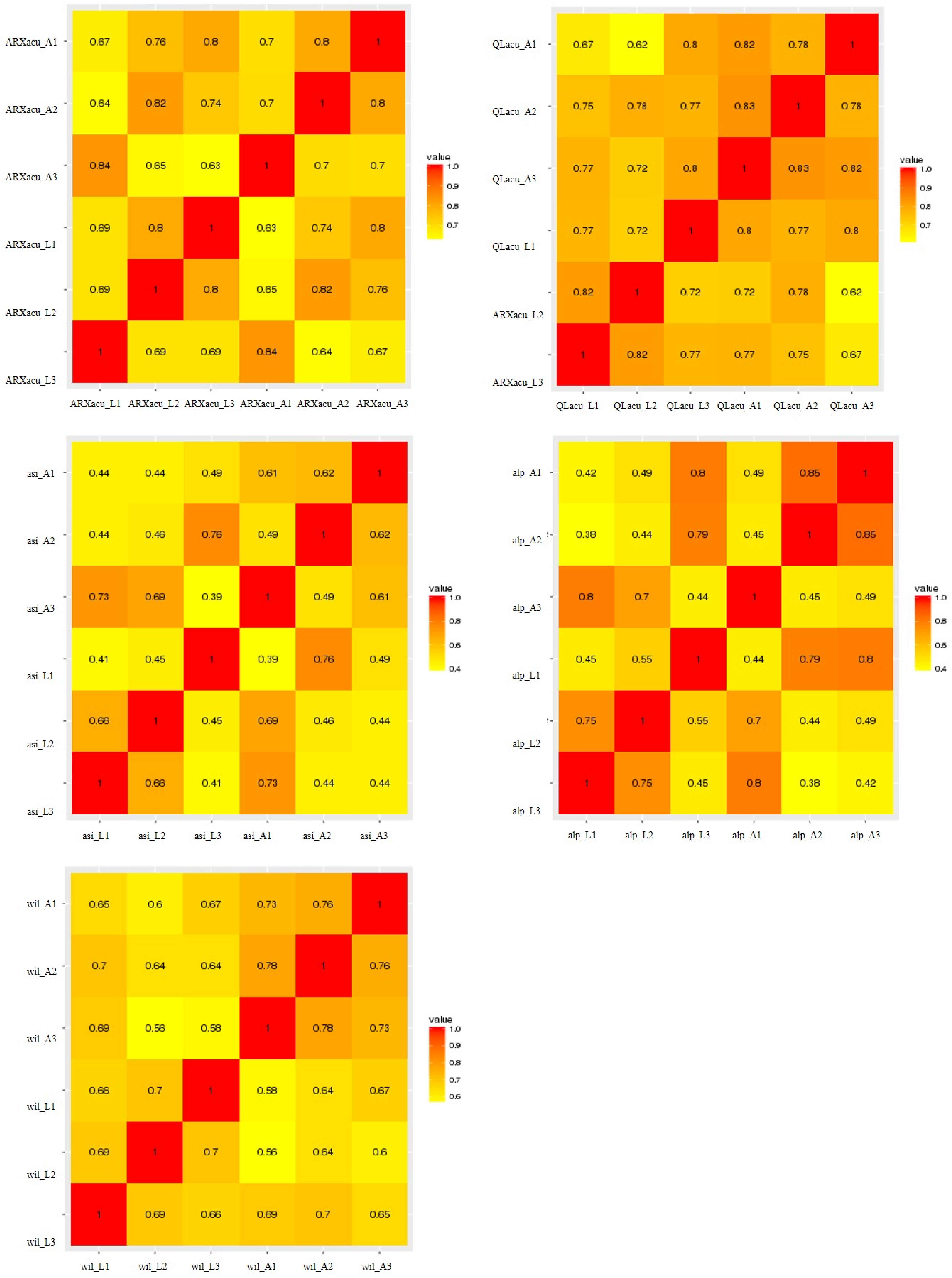

Figure S1 Correlation analysis of transcriptome data from different samples.
Abbreviations: acu, *Achillea acuminata*; asi, *A. asiatica*; alp, *A. alpina*; wil, *A. wilsoniana*; L, leaf tissue; A, stem apex.
