# Supplementary material for: Imprints of independent allopolyploid formations on patterns of gene expression in two sibling yarrow species (Achillea, Asteraceae)
Source: BMC Genomics. 2021 Apr 13;22:264. doi: 10.1186/s12864-021-07566-6 (PMC8045213; doi:10.1186/s12864-021-07566-6)
Supplement: Supplementary file 2 — Additional file 2: Supplementary Fig. S2. Patterns of gene expression detected by RT-qPCR to verify the RNA-Seq data. [file 12864_2021_7566_MOESM2_ESM.pdf]

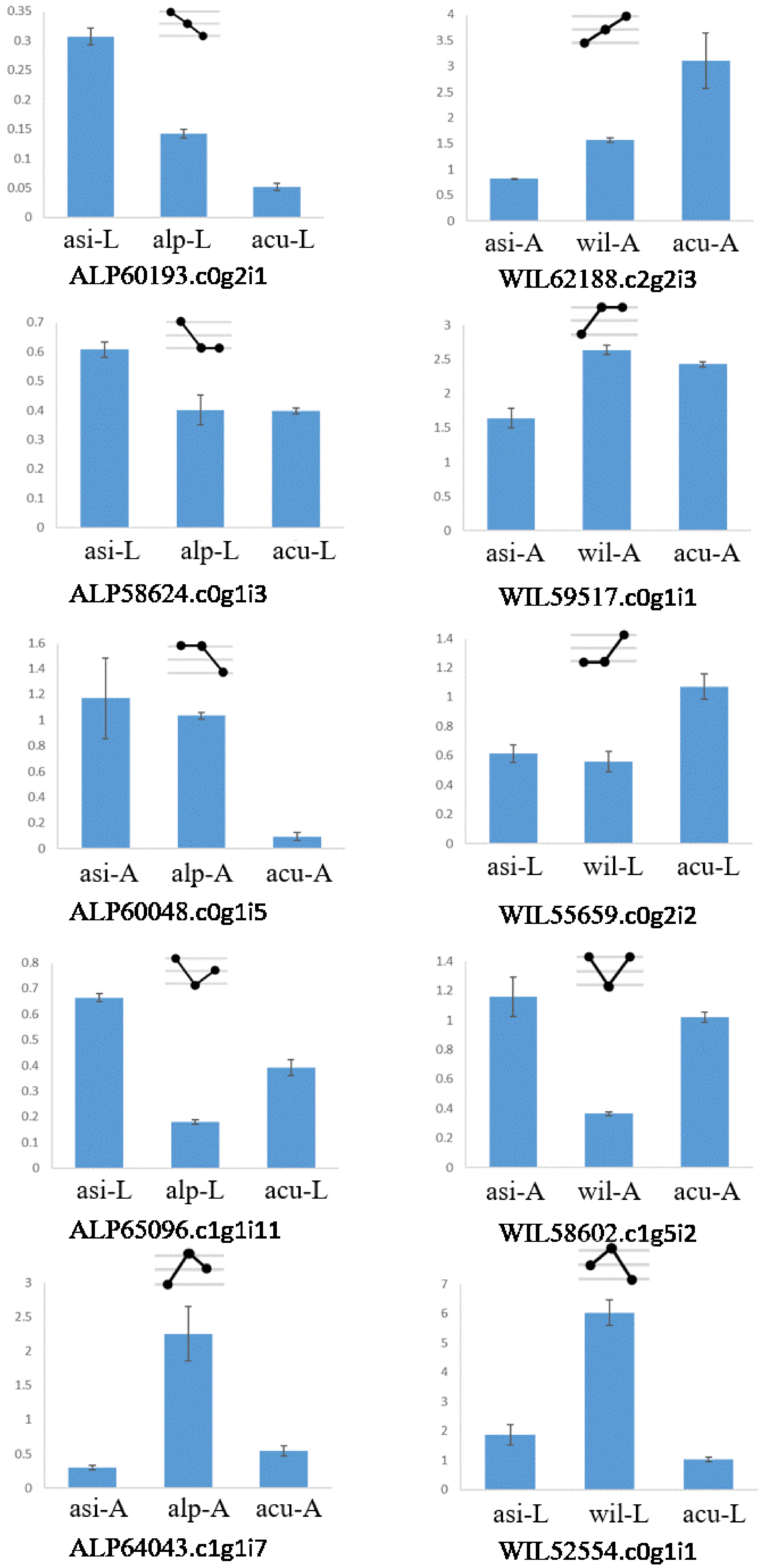

Figure S2 Patterns of gene expression detected by RT-qPCR to verify the RNA-Seq data. Each histogram shows the relative expression level of a gene among the allotetraploid and its parental species. Names of unigene of the allotetraploids are shown below the diagrams. The cartoon depiction above each diagram shows the expression inheritance category of this particular gene detected by RNA-Seq data. For the meaning of the carton depiction, readers are referred to Figure 3. Abbreviations: asi=*A. asiatica*; acu=*A. acuminata*; alp=*A. alpina*; wil=*A. wilsoniana*; A=stem apex; L=leaf tissue.
