# Supplementary material for: Imprints of independent allopolyploid formations on patterns of gene expression in two sibling yarrow species (Achillea, Asteraceae)
Source: BMC Genomics. 2021 Apr 13;22:264. doi: 10.1186/s12864-021-07566-6 (PMC8045213; doi:10.1186/s12864-021-07566-6)
Supplement: Supplementary file 3 — Additional file 3: Supplementary Table S1. Information of reads in the transcriptome data. [file 12864_2021_7566_MOESM3_ESM.pdf]

Table S1 Information of reads in the transcriptome data

Sample	Number of Raw Reads	Number of Bases	Proportion of Q20	Proportion of Q30	Avg. Quality	Number of Clean Reads	Proportion of Clean Reads
acuARX_A1_R1	20567641	2077331741	98.01%	96.58%	38.41	19754566	96.05%
acuARX_A1_R2	20567641	2077331741	98.71%	97.69%	38.7	19754566	96.05%
acuARX_A2_R1	20279758	2048255558	97.72%	96.12%	38.25	19346067	95.40%
acuARX_A2_R2	20279758	2048255558	98.51%	97.38%	38.56	19346067	95.40%
acuARX_A3_R1	18499630	1868462630	98.03%	96.70%	38.47	17608441	95.18%
acuARX_A3_R2	18499630	1868462630	98.83%	97.93%	38.79	17608441	95.18%
acuARX_L1_R1	22109467	2233056167	98.14%	96.80%	38.46	21268073	96.19%
acuARX_L1_R2	22109467	2233056167	98.72%	97.69%	38.7	21268073	96.19%
acuARX_L2_R1	18339241	1852263341	97.84%	96.35%	38.31	17459292	95.20%
acuARX_L2_R2	18339241	1852263341	98.61%	97.50%	38.59	17459292	95.20%
acuARX_L3_R1	18709676	1889677276	98.31%	97.13%	38.58	17858116	95.45%
acuARX_L3_R2	18709676	1889677276	98.84%	97.94%	38.79	17858116	95.45%
acuQL_A1_R1	20284407	2048725107	98.54%	97.41%	38.59	19236897	94.84%
acuQL_A1_R2	20284407	2048725107	97.02%	94.79%	37.81	19236897	94.84%
acuQL_A2_R1	19486101	1968096201	98.53%	97.40%	38.58	18553443	95.21%
acuQL_A2_R2	19486101	1968096201	97.18%	95.09%	37.9	18553443	95.21%
acuQL_A3_R1	19251783	1944430083	98.77%	97.74%	38.71	18554269	96.38%
acuQL_A3_R2	19251783	1944430083	97.98%	96.55%	38.36	18554269	96.38%
acuQL_L1_R1	19034919	1922526819	98.49%	97.33%	38.56	18060589	94.88%
acuQL_L1_R2	19034919	1922526819	97.18%	95.09%	37.89	18060589	94.88%
acuQL_L2_R1	18567803	1875348103	98.45%	97.25%	38.53	17562422	94.59%
acuQL_L2_R2	18567803	1875348103	96.92%	94.63%	37.74	17562422	94.59%
acuQL_L3_R1	17016388	1718655188	98.82%	97.83%	38.75	16331934	95.98%
acuQL_L3_R2	17016388	1718655188	97.71%	96.12%	38.23	16331934	95.98%
asi_A1_R1	24563354	2480898754	97.77%	96.23%	38.34	23501958	95.68%
asi_A1_R2	24563354	2480898754	98.57%	97.54%	38.66	23501958	95.68%
asi_A2_R1	21312464	2152558864	97.61%	96.00%	38.22	20220013	94.87%
asi_A2_R2	21312464	2152558864	98.60%	97.51%	38.59	20220013	94.87%
asi_A3_R1	19011564	1920167964	98.34%	97.18%	38.59	18210777	95.79%
asi_A3_R2	19011564	1920167964	98.83%	97.92%	38.78	18210777	95.79%
asi_L1_R1	22816575	2304474075	98.07%	96.69%	38.44	21950055	96.20%
asi_L1_R2	22816575	2304474075	98.62%	97.56%	38.65	21950055	96.20%
asi_L2_R1	20865478	2107413278	98.15%	96.82%	38.46	20038013	96.03%
asi_L2_R2	20865478	2107413278	98.64%	97.58%	38.65	20038013	96.03%
asi_L3_R1	19363845	1955748345	97.82%	96.32%	38.31	18434577	95.20%
asi_L3_R2	19363845	1955748345	98.63%	97.54%	38.6	18434577	95.20%
alp_A1_R1	20924099	2113333999	98.74%	97.73%	38.72	20073140	95.93%
alp_A1_R2	20924099	2113333999	98.21%	96.91%	38.51	20073140	95.93%
alp_A2_R1	21292753	2150568053	98.60%	97.38%	38.58	20370264	95.67%

alp_A2_R2	21292753	2150568053	97.61%	95.84%	38.15	20370264	95.67%
alp_A3_R1	22198521	2242050621	98.61%	97.40%	38.59	21383720	96.33%
alp_A3_R2	22198521	2242050621	97.94%	96.40%	38.34	21383720	96.33%
alp_L1_R1	22716578	2294374378	98.71%	97.69%	38.7	21755043	95.77%
alp_L1_R2	22716578	2294374378	98.25%	97.01%	38.54	21755043	95.77%
alp_L2_R1	19169276	1936096876	98.82%	97.90%	38.77	17940918	93.59%
alp_L2_R2	19169276	1936096876	98.00%	96.62%	38.44	17940918	93.59%
alp_L3_R1	22587729	2281360629	98.60%	97.38%	38.58	21581867	95.55%
alp_L3_R2	22587729	2281360629	97.90%	96.34%	38.32	21581867	95.55%
wil_A1_R1	20609677	2081577377	98.65%	97.62%	38.68	19752057	95.84%
wil_A1_R2	20609677	2081577377	98.06%	96.68%	38.45	19752057	95.84%
wil_A2_R1	23019622	2324981822	98.55%	97.45%	38.59	21857180	94.95%
wil_A2_R2	23019622	2324981822	97.70%	96.11%	38.25	21857180	94.95%
wil_A3_R1	19266348	1945901148	98.82%	97.91%	38.77	18146451	94.19%
wil_A3_R2	19266348	1945901148	98.23%	96.98%	38.52	18146451	94.19%
wil_L1_R1	22422769	2264699669	98.68%	97.64%	38.68	21529819	96.02%
wil_L1_R2	22422769	2264699669	98.15%	96.81%	38.48	21529819	96.02%
wil_L2_R1	20915956	2112511556	98.54%	97.42%	38.57	19917188	95.22%
wil_L2_R2	20915956	2112511556	97.82%	96.28%	38.29	19917188	95.22%
wil_L3_R1	20471089	2067579989	98.81%	97.90%	38.77	19075706	93.18%
wil_L3_R2	20471089	2067579989	98.18%	96.92%	38.51	19075706	93.18%

Abbreviations: acuARX, Arxan population of *A. acuminata*; acuQL, Qinling population of *A. acuminata*; alp, *A. alpina*; asi, *A. asiatica*; wil, *A. wilsoniana*; A, stem apex; L, leaf tissue.
