# Supplementary material for: Imprints of independent allopolyploid formations on patterns of gene expression in two sibling yarrow species (Achillea, Asteraceae)
Source: BMC Genomics. 2021 Apr 13;22:264. doi: 10.1186/s12864-021-07566-6 (PMC8045213; doi:10.1186/s12864-021-07566-6)
Supplement: Supplementary file 4 — Additional file 4: Supplementary Table S2. Relative expression level and expression bias of homeologs in the studied Achillea allotetraploid species. [file 12864_2021_7566_MOESM4_ESM.pdf]

Table S2 Relative expression level and expression bias of homeologs in the studied *Achillea* allotetraploid species

Relative expression level of homeologs	No. of genes with higher expression level of the *A. acuminanta*-homeolog				No. of genes with equal expression level of the two homeologs				No. of genes with higher expression level of the *A. asiatica*-homeolog			
Expression bias	Expression bias towards *A. acuminata*	No expression bias	Expression bias towards *A. asiatica*	Total	Expression bias towards *A. acuminata*	No expression bias	Expression bias towards *A. asiatica*	Total	Expression bias towards *A. acuminata*	No expression bias	Expression bias towards *A. asiatica*	Total
A. alpina (stem apex)	764	439	20	1223	838	4418	1191	6447	10	349	1037	1396
A. alpina (leaf)	414	400	21	835	471	3689	1672	5832	4	168	861	1033
A. wilsoniana (stem apex)	715	569	25	1309	848	4235	1401	6484	20	372	1032	1424
A. wilsoniana (leaf)	456	559	23	1038	471	3172	1438	5081	17	239	823	1079
