# Supplementary material for: Imprints of independent allopolyploid formations on patterns of gene expression in two sibling yarrow species (Achillea, Asteraceae)
Source: BMC Genomics. 2021 Apr 13;22:264. doi: 10.1186/s12864-021-07566-6 (PMC8045213; doi:10.1186/s12864-021-07566-6)
Supplement: Supplementary file 5 — Additional file 5: Supplementary Table S3. Primers used in RT-qPCR assays. [file 12864_2021_7566_MOESM5_ESM.pdf]

Table S3 Primers used in RT-qPCR assays

Primer name	Primer sequence（5’– 3’）
AchG6PDH	F: GACTTTGGTACAGAAGGACGAG<br>R: CCAACGACTACATCTTCGAGTT
AchPP2A	F: GCGTTTACCCGTGGTAGTAA<br>R: GTCGACAATGTTGAAGCTTTGAT
ALP60193.c0g2i1	F: TCTTGGCACCCACAATGATTTA<br>R: CAGTCACTTCCCTCACTGTTAC
ALP58624.c0g1i3	F: CACCACATTTGCGCACTTAC<br>R: ACCCATGAGACTGTTGTCTTG
ALP60048.c0g1i5	F: ACGCGAACCCTTCACAAA<br>R: ACTTCGAGTTTCCTCCCAATG
ALP65096.c1g1i11	F: CGATTAAGTTGAGGAGGGAGAAG<br>R: CGTCCACTTGAGACGCATTA
ALP64043.c1g1i7	F: GTCCCACCAGAATCTGGTTAAT<br>R: GCAAGTTCTGGATATCCTCTCC
WIL62188.c2g2i3	F: GTTCCCGTCCATCGTAGATT<br>R: TGCTTGGGTAAAGGATTATTAGTTG
WIL59517.c0g1i1	F: CCTGATGGGAAACGGACTAAT<br>R: CTTCACCACCAGGTACCATAAG
WIL55659.c0g2i2	F: GTTCAAGAAAGCCTGTGGAAAG<br>R: GCGTCCTGTTGATGGGAATA
WIL58602.c1g5i2	F: GGTGGTAATAGTCTTGGCTGTC<br>R: CGACTCCAAAGTAAGGGAACTG
WIL52554.c0g1i1	F: GGAGCAGGTTGAAGTGATGAA<br>R: AGGCGACATATATAGGGTCTGA
